# Formula Optimization for In Vitro Assessment of Nutritional, Sensory, and Bioactive Properties of Functional Bee Product Mixtures

**DOI:** 10.1002/fsn3.70151

**Published:** 2025-04-15

**Authors:** Melike Ağirsaygin, Müge Hendek Ertop

**Affiliations:** ^1^ Institute of Science, Department of Food Engineering Kastamonu University Kastamonu Türkiye; ^2^ Faculty of Engineering and Architecture, Department of Food Engineering Kastamonu University Kastamonu Türkiye

**Keywords:** bee product mixture, bioactivity, optimization, response surface methodology

## Abstract

The bee products, whose rich bioactive and nutritional properties are supported by substantial scientific research, present significant and safe alternatives for today's consumers' daily diets. Considering the challenges today's individuals face in accessing safe and high‐quality bee products, as well as their expectation for foods that are not only satiating but also functional, offering these products as a single mixture presents a promising alternative. This study aimed to develop a bee product mixture (BPM) by utilizing three primary bee products (two types of honey [multifloral and chestnut], bee‐collected pollen, and propolis). The formulation was optimized using the response surface methodology, focusing on sensory acceptability and bioactive properties. The optimized levels for BPM with multifloral honey were determined as 1.73% propolis, 10.24% pollen, and 88.03% honey, and for BPM with chestnut honey, were determined as 4.07% propolis, 10.24% pollen, and 85.69% honey. Both BPMs were compared regarding physicochemical and bioactive properties, amino acid, and mineral compositions. They demonstrated significantly higher phenolic content, amino acid composition, and mineral profiles than the two types of honey used as raw materials (chestnut and multifloral honey). Although BPM with chestnut honey exhibited higher values, it was revealed that multifloral honey could be transformed into a nutritionally value‐added product with the bee products incorporated in it. The pollen was found to be the primary bee product that determined the functional properties of BPM. While the study results, focused on the optimization and fundamental analysis of mixtures, provide general insights into their functionality, further advanced clinical studies are necessary to draw definitive conclusions regarding their apitherapeutic properties.

## Graphical abstract

This study aimed to develop a bee product mixture (BPM) by utilizing three primary bee products (two types of honey [multifloral and chestnut], pollen, and propolis). The formulation was optimized using the response surface methodology, focusing on sensory acceptability and bioactive properties. The optimized bee product mixtures demonstrated significantly higher phenolic content, amino acid composition, and mineral profiles compared to the two types of honey used as raw materials (chestnut and multifloral honey).

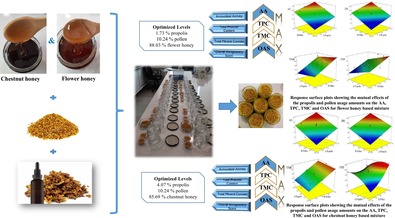

**Abbreviations**
AA: antioxidant activity
ANOVA: analysis of variance
AOAC: association of official analytical chemists
CAPE: caffeic acid phenyl ester
CCRD: the central composite rotatable design
DPPH: 1,1‐Diphenyl‐2‐picrylhydrazyl
GAE: gallic acid equivalent
HPLC: high pressure liquid chromatography
ICP–OES: inductively coupled plasma–optical emission spectrometry
LC–MS/MS: liquid chromatography/mass spectrometry
RSM: response surface methodology
TMC: total mineral content
TPC: total phenolic content
UV: ultraviolet

## 1 Introduction

Today's fast‐paced lifestyle, inadequate nutritional diversity, and fatigue from work and social life have become prevalent issues. To address these challenges, interest in dietary supplements and functional foods is growing steadily, focusing on achieving sustainable good health and well‐being. Consumers increasingly prefer to incorporate foods into their daily diets that not only provide satiety but also combine various functional properties. From this perspective, bee products, whose rich bioactive and nutritional properties are supported by substantial scientific research, offer significant and reliable alternatives to today's consumers. Studies investigating the physicochemical properties of bee product mixtures are limited, as most research primarily focuses on incorporating honey, pollen, propolis, or royal jelly into various food matrices rather than exploring their combined use.

Propolis is a bee product with resinous material collected from the exudates and buds of plants mixed with pollen, wax, and bee enzymes. It is known to be used in complementary medicine because of its anti‐inflammatory, antimicrobial, immunomodulatory, antioxidant, and immunostimulatory activities from ancient times (Mele 2023; Bankova et al. 2021; Korkmaz et al. 2021; Asem et al. 2020). The propolis content, which is rich in bioactive compounds, varies depending on its botanical and geographical origin and harvest time (Kasote et al. 2022). Overall, more than 300 different bioactive compounds, such as phenolic aldehydes, ketones, and polyphenols (phenolic acids, flavonoids, and esters), have been identified in propolis. The flavonoid group includes chrysin, pinocembrin, apigenin, galangin, kaempferol, quercetin, tectocrisin, and pinostrobin, among others (El‐Guendouz et al. 2019). Another important group of identified bioactive compounds comprises phenolic acids, which also exhibit aromatic properties; these compounds include caffeic, cinnamic, ferulic, benzoic, p‐coumaric, and salicylic acids (Do Nascimento Araújo et al. 2020).

The pollen content and diversity, especially dominant and secondary pollen groups, are among the most crucial factors determining the quality and composition of honey. Monofloral honey types primarily originate from a single botanical source, and the dominant plant species' nectar and pollen significantly influence honey's sensory properties. Some monofloral honey types, such as chestnut honey, have been reported to benefit human health (Alkan 2020). Due to their antibacterial and anti‐inflammatory properties, they can be used for wound treatment, certain diseases, and apitherapeutic purposes. Consequently, interest in the melissopalynological verification of the botanical origin of honey has been increasing in recent years (Jandrić et al. 2015). Depending on its botanical origin, honey, a natural product of honeybees, serves as a functional food with protective and therapeutic benefits against numerous diseases, thanks to its rich content of vitamins, minerals, organic acids, and enzymes (Erdem et al. 2024; Dashora et al. 2011). It contains several major (calcium (Ca), potassium (K), phosphorus (P), magnesium (Mg)) and trace elements ((Fe), copper (Cu), zinc (Zn), selenium (Se), fluorine (F), and chlorine (Cl)) (Mayda et al. 2020). In terms of mineral composition, chestnut honey has been found to have higher concentrations of potassium, calcium, and manganese compared to other types of honey (Kolaylı et al. 2008; Küçük et al. 2007). This rich content makes honey a versatile natural product with many health benefits (Sajtos et al. 2019; Solayman et al. 2016). Honey plays a key role in treating various ailments, such as ulcers, stomach disorders, palpitations, heart failure, cough, bone conditions, allergies, anemia, bronchitis, skin issues, throat pain, and nervous system disorders. Additionally, it helps alleviate constipation, strengthens the heart, enhances blood circulation, aids in fat digestion, and promotes healing burns and wounds (Molan and Cooper 2000).

Bee‐collected pollen is the agglutination product, composed of pollen grains collected by worker bees, held together with nectar and/or honey and salivary secretions, and gathered at the entrance of the hive (El Ghouizi et al. 2023). Bee‐collected pollen is a nutrient‐rich substance consisting of carbohydrates (13%–55%), proteins (10%–40%), lipids (1%–13%), crude fiber (0.3%–20%), and ash content (2%–6%) (Campos et al. 2008). It also contains all essential amino and fatty acids, free amino acids, B‐complex vitamins, vital minerals, carotenoids, and flavonoids. It was reported that polyphenolic compounds in bee pollen protect from biotic (microbial growth) and abiotic (synthesis of reactive oxygen species, UV radiation, and high temperature) stress by their capacity to neutralize free radicals (Thakur and Nanda 2020). Renowned for its remarkable nutritional value and therapeutic benefits, bee‐collected pollen is widely used today as a dietary supplement.

The superior nutritional qualities of each bee product individually, such as pollen, which is considered a “whole food,” and propolis, known for its rich polyphenolic content and effectiveness as a robust immune system support, have been demonstrated in many previous studies. However, there are not enough studies on the synergistic properties (such as antioxidant and antimicrobial), functional (such as enriching the nutritional profile, prebiotic properties), and potential benefits (strengthening the immune system, enhancing anti‐inflammatory properties or ease of use/consumption) of the mixtures to be formed by combining these products. A limited number of studies have shown that the combination of honey, propolis, and pollen offers synergistic benefits, such as enhanced antioxidant, antimicrobial, and hepatoprotective effects (Kamel et al. 2023; Münstedt and Männle 2019; Saral et al. 2016). A few studies highlight not only honey bee products but also their combination with different raw materials, such as yogurt and medicinal and aromatic plants, which have demonstrated significantly enhanced pharmacological, nutritional, and synergistic effects when combined compared to their individual applications (Kamel et al. 2023; Soylu and Bayram 2020; Abdelmonem et al. 2012; Boukraa 2008). The synergistic interactions among bioactive compounds in natural products can substantially amplify their biological effectiveness.

Furthermore, considering today's individuals' difficulty accessing safe and high‐quality bee products, offering these products as a single mixture is a promising approach. This study aimed to develop a bee product mixture by utilizing three primary bee products (two types of honey, bee‐collected pollen, and propolis). The formulation was optimized using the response surface methodology, focusing on sensory acceptability and bioactive properties. The bee product mixtures produced by optimization were evaluated in terms of some physicochemical, bioactive properties, and nutritional contents.

## 2 Materials and Methods

### 2.1 Samples

Chestnut honey was obtained from the apiaries of the Doğanyurt districts, which include the borders of the Kastamonu chestnut forest region, during the 2023 harvest period. Because it contains over 90% *Castanea sativa* Miller, this sample was determined to be chestnut honey as a monofloral honey type. The multifloral honey was supplied from the Bitlis region (Tulliana Beekeeping Company). According to melissopalynological analysis, the pollen types of the honey sample were determined as Fabaceae, Asteraceae, Brassicaceae, Rosaceae, Lamiaceae, and Boraginaceae, and identified as a multifloral honey. The propolis and bee‐collected pollen samples were purchased from Beeonfood Food Limited Company. Propolis was supplied in extract form containing 20% raw propolis, with glycerin as the final solvent, and in an amber colored bottle.

### 2.2 Chemicals

1,1‐Diphenyl‐2‐picrylhydrazyl (DPPH) and Folin–Ciocalteu's reagent were obtained from Sigma‐Aldrich Co. (Munich, Germany). Acetonitrile, sodium carbonate, ethanol, methanol, and phenolic standards were supplied by Merck KGaA (Darmstadt, Germany).

### 2.3 Melissopalynological Analysis of Samples

The pollen content of the samples was determined using melissopalynological analysis according to the methodology described by Louveaux et al. (1978), with some modification. Five grams of sample was mixed with a glass rod and transferred into a test tube, followed by the addition of 10 mL of distilled water. To facilitate dissolution, the test tubes were placed in a water bath at approximately 45°C for 30–45 min. The samples were then centrifuged at 3500 rpm for 45 min, and the supernatant was discarded. A portion of the sediment remaining at the bottom of the tube was taken, and a small amount of basic fuchsin was added. To ensure the dissolution of the basic fuchsin, the sample was heated at 30°C–40°C and infused with glycerin–gelatin. This material was then transferred onto a slide, covered with an 18 × 18 mm coverslip, and examined under a light microscope (Nikon Eclipse E100).

### 2.4 Optimization of the Formulations

This study comprised three stages: the optimization of the bee product mixture formulation based on the experimental design, validation of the optimization results, and characterization of the optimized mix products. Firstly, according to the experimental design suggested by the software, 13 mixture samples were prepared, AA, TPC, and TMC analyses were performed individually, and after evaluation of the results by the software, the optimized formulation for multifloral and chestnut honey was obtained. Secondly, their validation was carried out, and then thirdly, the analyses of raw materials and mixtures are given in tables comparatively. In other words, the 13 mixtures suggested by the software (a total of 26 mixtures were prepared for two types of honey) were reduced to two individual mixtures.

#### 2.4.1 Experimental Design

Two types of mixes were prepared: one based on multifloral honey and the other on chestnut honey. This study considered the honey, pollen, and propolis rate % in the formula as three independent factors. Antioxidant activity (AA) (inhibition %), total phenolic content (TPC) (mg GAE/100 mL), total mineral content (TMC %), and overall acceptability score were the four main responses. Response surface methodology (RSM)–The central composite rotatable design (CCRD) desirability function was used to evaluate the effect of the three independent factors on the four responses (Design Expert 7.0.0, Stat‐Ease Inc., Minneapolis, MN, USA). The experimental design involved 13 design points for the multifloral honey and chestnut honey–based mix, including five replicates of the central point. Probability values (P) at a 95% confidence level were used to determine the significance and effectiveness of the response. Lack of fit test and analysis of variance (ANOVA) were carried out using software. For the formula optimization, the minimum and maximum amounts of honey, pollen, and propolis were entered into the Design Expert software (7.0.0, Stat‐Ease Inc., Minneapolis, MN, USA), and all experimental levels to be used in the formulation were generated by the software. The experimental levels of factors for honey concentration (%) in the mix formula were as follows: 85.49, 89.03, 91.50, 93.97, 97.51; for pollen in the formula: 0, 1.76, 6.0, 10.24, 12.00; and for propolis in the formula: 0, 0.73, 2.50, 4.27, 5.00. The experimental design was carried out for two types of honey‐based mixing. According to the experimental design of each mixing formula was applied. The granule structure of the bee pollen was reduced using a porcelain mortar before being used in the mixture. For the 13 mixture formulas determined in the experimental design, honey, pollen, and propolis were weighed and mixed using a mixer for about 10 min at room temperature until a homogeneous mixture was achieved. The mixtures obtained were filled into glass jars and their lids were sealed. The mixtures were kept at room temperature in the dark until analysis.

#### 2.4.2 Validation of the Optimization Results

The validation was carried out by preparing the bee product mix in triplicate according to optimal conditions for each type of honey. The average values of the responses were calculated. The estimated values from the model were compared with the actual averages using a one‐sample *t*‐test. The lack of a statistically significant difference (*p* > 0.05) between the results from the validation test indicates that the model developed through optimization was experimentally successful.

#### 2.4.3 Characterization of the Optimized Products

##### 2.4.3.1 Physicochemical Properties

Moisture, ash, fat, and protein (%) content of the mixture samples and bee products were evaluated by following the standard analytical methods. All the mentioned parameters were assessed by using AOAC methods (2000). To determine the dry matter content of the mixture samples, an Abbe refractometer was used, and measurements were conducted at an ambient temperature of 20°C. To measure pH, the sample was homogenized with distilled water (1:9, w/v) by an Ultra‐Turrax (T25, IKA, Königswinter, Germany) and the pH value was measured (ST3000, Ohaus, USA). The color profile of the samples was determined on five different points using a colorimeter (NR145, 3nh, China) as L*, a*, and b*. Mean values were then calculated (Hayta and Hendek Ertop 2019).

##### 2.4.3.2 Total Phenolic Content (TPC)

TPC of the samples was determined according to the Folin–Ciocalteu assay (Shahidi and Naczk 1995). Firstly, the bee products and their mixtures, 2 g of sample, were extracted with 20 mL of 80% ethanol. The obtained extract was then diluted at a ratio of 1:10 with 80% ethanol, while the propolis extract was diluted at 1:100 before being analyzed. Briefly, 0.15 mL of Folin–Ciocalteu's reagent (0.2 N) was added to 0.15 mL of extract. Then, mixing the tube using a vortex, 0.12 mL of Na_2_CO_3_ (7.5%) solution and 1.2 mL of distilled water were added to the reaction mixture. The absorbance readings were taken at 760 nm after incubation at room temperature for 1 h using a UV–VIS spectrophotometer (Shimadzu Corporation, Japan) against the blank prepared using ethanol instead of extracts. Results were expressed as gallic acid equivalent (μg GAE/g) using the calibration curve [Concentration = (Abs + 0.020)/0.009] obtained using gallic acid standard solutions.

##### 2.4.3.3 Antioxidant Activity

The antioxidant activity (inhibition %) of the bee products, propolis extracts, and mixture samples was determined using the 2,2‐diphenyl‐1‐picrylhydrazyl (DPPH) radical scavenging procedure. A quantity of 4900 μL of DPPH (0.025 g/L ethanol) solution and 1000‐μL ethanol (80%) were added to 300 μL of diluted (100 times) extracts, and the mixture was vortexed. After the mixture was incubated for 30 min, the absorbance readings were taken at 517 nm using a UV–VIS spectrophotometer. Inhibition % value was calculated with the following equation.

(1) $$\text{Inhibition}\%=\left[1-\left(\frac{\mathrm{Abs}\;\text{Sample}}{\mathrm{Abs}\;\text{Control}}\right)\right]*100$$

##### 2.4.3.4 Phenolic Content

The phenolic components of the extracts were analyzed on a LC–MS/MS device (LCMS‐8030 Plus, Shimadzu LC–MS/MS, Japan). Inertsil ODS4 3 μM, 2,1 × 50 mm column (Agilent Technologies) was utilized in the analysis. Deionized water (A) containing 0.1‰ formic acid and methanol (B) containing 0.1‰ formic acid were used as the mobile phases. The mobile phase program was as follows: the gradient was set 5:95 A:B in the range of 0–4 min, 5:95 A:B in the range of 4–7 min, 95:5 A:B in the range of 7,01–12 min. The mobile phase flow rate was set at 0.4 mL/min. The column was kept at 40°C and the injection volume was set at 10 μL throughout the study.

##### 2.4.3.5 Amino Acid Content

A sample of 0.5 g was burned with 20 mL of HCl at 110°C for 18–24 h. Twenty milliliters of deionized water was added and dried in an evaporator at 70°C. It was completed to 25 mL in a volumetric flask with deionized water. The amino acid contents of the extracts were analyzed on an LC–MS/MS device (LCMS‐8030 Plus, Shimadzu LC–MS/MS, Japan). A Zorbax Eclipse AAA 4,6 X 150 mm; 3,5 μm column (Agilent Technologies) was utilized in the analysis. Deionized water (A) containing 0.1‰ formic acid and methanol (B) containing 0.1‰ formic acid were used as the mobile phases. The mobile phase program was as follows: the gradient was set 85:15 A:B in the range of 0–2.5 min, 70:30 A:B at 4.6 min, 60:40 A:B at 7.5 min, 30:70 A:B at 9.70 min, 0:100 A:B at 10.50 min, and at 13.50 min, 85:15 A:B at 14 min. The mobile phase flow rate was set at 1 mL/min. The column was kept at 40°C, and the injection volume was set at 0.2 μL throughout the study.

##### 2.4.3.6 Mineral Contents

The mineral content of the samples was assessed using a microwave‐assisted HNO_3_ digestion procedure (CEM MARS6, USA) followed by analysis with inductively coupled plasma–optical emission spectrometry (ICP–OES) (Spectro Blue, Germany). Approximately 0.25 g of each sample was mixed with 10 mL of HNO_3_ (65% v/v) in PTFE flasks. The digestion program included heating to 200°C for 15 min, followed by a 15‐min hold at 200°C. After cooling to room temperature, the solutions were transferred to 25‐mL polyethylene flasks and diluted with ultrapure water. The digested samples were filtered through microfilters and analyzed by ICP–OES (Al Khalifa and Ahmad 2010). Calibration standards were prepared using a multielement standard stock solution (Merck, Germany). All measurements were performed in triplicate.

##### 2.4.3.7 Sensory Analysis

The sensory evaluation of the mixture samples prepared according to the trial design was conducted by assessing appearance/color, consistency, taste, flavor, mouthfeel, and overall acceptability. Each sample was coded with three‐digit numbers and served to panelists in plain glasses, with one spoonful per portion. Panelists were selected based on specific criteria: individuals who had previously consumed bee products, were not allergic to them, and were non‐smokers. Prior to the evaluation, the panelists were informed about the evaluation criteria and the scoring system. A hedonic scale ranging from 1 (I did not like it at all) to 5 (I liked it very much) was used for scoring the sensory properties. The overall acceptability score was calculated as the average of all individual scores, providing a comprehensive measure of panelists' preferences.

### 2.5 Statistical Analysis

Analysis of variance (ANOVA) (IBM SPSS 1.0.0.781) was used to evaluate the results statistically. After optimization, the obtained results were validated experimentally. For comparison (*p* < 0.05) of the results, a one‐sample *t‐*test (SPSS 17.0.1, Chicago, IL, USA) was used. The data were given as the mean ± standard deviation.

## 3 Results

### 3.1 Optimization of Formulation Parameters

The software subsequently analyzed the results of the antioxidant activity (AA), total phenolic content (TPC), total mineral content (TMC), and overall acceptability score (OAS) values based on the experimental design. “Sequential model sum of squares” and “Lack of fit” tests were performed (Table 1) for the results. R‐squared (R^2^), adjusted R^2^, and the standard deviation were calculated for each function. After comparing the values subsequently, the suggested functions were ascertained.

**TABLE 1:** Statistical parameters of optimization; *p* values for model selection and lack of fit tests; model and independent variable factors; variance analysis results of functions (a); Selected solutions determined by desirability function and comparison of the results obtained from optimum point verification tests with the estimated values from model (b).

		Multifloral honey				Chestnut honey			
		AA	TPC	TMC	OAS	AA	TPC	TMC	OAS
Model selection and lack of fit test	Quadratic	0.3483	0.7023	0.3459	0.3630	0.2932	0,5863	0.8519	**0.0111**
	Linear	**< 0,0001**	**< 0,0001**	**0.0001**	**0.0072**	**0.0002**	**< 0.0001**	**< 0.0001**	0.0385
	Cubic	0.1901	0.1387	0.0484	0.0900	0.2802	0.5410	00192	0.2913
	Lack of fit	**0.3602**	**0,4179**	**0.1262**	**0.1015**	**0,2193**	**0,7154**	**0,0912**	**0.3609**
Model and independent variable factors	Model	< 0.0001	< 0,0001	0.0001	0,0072	0,0002	< 0,0001	< 0.0001	0.0026
	A‐Propolis	**0.0002**	**0.0007**	0.0771	**0,0227**	**0.0020**	**< 0,0001**	0.4835	**0,1289**
	B‐Pollen	**< 0.0001**	**< 0,0001**	**< 0.0001**	**0.0114**	**0.0003**	**0.0002**	**< 0.0001**	**0.0010**
	AB	—	—	—	—	—	—	—	**0,0182**
	A^2^	—	—	—	—	—	—	—	**0,0145**
	B^2^	—	—	—	—	—	—	—	**0.0497**

	Intercept	+5.021	+312,405	+0,114	+4661	+22.212	+418,392	+0,466	+4,030
	A	+6.649	+56,485	+0,015	−0.122	+6504	+96,795	+0.006	−0.108
	B	+4619	+34,006	+0,022	−0.059	+3580	+31.975	+0,038	−0,059
	AB	—	—	—	—	—	—	—	+0,014
	A^2^	—	—	—	—	—	—	—	−0,027
B^2^	—	—	—	—	—	—	—	+0.003
*R* ^2^		0.938	0.876	0.835	0.627	0.825	0.894	0.916	0.895
Adjusted *R* ^2^		0.926	0,852	0.802	0.553	0.790	0.873	0.899	0.820
Variation coefficient (%)		10.77	8.96	13.59	5,68	13.08	7.89	6.15	1.95
**(a)**									
**Honey type**		**Independent factors (%)**			**AA (%)**	**TPC (mg/100 mL)**	**TMC (%)**	**OAS**	
		**A (Propolis)**	**B (Polen)**	**C (Honey)**					
Multifloral		1.73	10.24	8803	62.11	758.98	0.364	3.85	
Chestnut		4.07	10.24	85.69	85.35	1139.84	0.878	3.73	
**(b)**									
**Honey type**		**Response**	**Estimated value**	**Average experimental result**	**Difference**	***p*** *			
Multifloral		AA	62.11	66.34 ± 3,03	+4.23	0.132			
		TPC	758.98	780.33 ± 10.28	+21.35	0.727			
		TMC	0.364	0.443 ± 0,05	+0.079	0.112			
		OAS	3.85	3.84 ± 0.05	−0.01	0.053			
Chestnut		AA	85.35	86.30 ± 0.40	+0,95	0.053			
		TPC	1139.84	1215.33 ± 99.08	+75.49	0.318			
		TMC	0.878	0.870 ± 0.02	−0.008	0.536			
		OAS	3.73	3.62 ± 0.16	−0.11	0,346			

*Note:* *, The values *p* < 0.05 are statistically significant.

Abbreviations: AA, antioxidant activity; OAS, overall acceptability score; TMC, total mineral content; TPC, total phenolic content.

The linear function was accepted for AA, TPC, TMC, and OAS for the multifloral honey mixture (*p* < 0.05). While the linear function was accepted for AA, TPC, and TMC, the quadratic function was accepted for OAS for the chestnut honey mixture (*p* < 0.05). “Lack of fit” was insignificant (*p* > 0.05) for four responses to both mixture types. The influence of the propolis usage amount, which was one of the independent factors on the TPC, AA, and OAS, was statistically significant (*p* < 0.05). However, it was insignificant on TMC for both mixtures. However, the influence of pollen usage amount had a statistically significant (*p* < 0.05) effect on all independent factors for both mixture types. The model was approved to be statistically significant (*p* < 0.05) for all the extraction types and responses. The results of the ANOVA for the selected function are presented in Table 2. Final equations were coded with the following factors:

**TABLE 2:** Some physicochemical properties of raw materials and optimized bee product mixtures (BPM).

		Chestnut honey	Multifloral honey	Propolis	Pollen
**(a)**					
Antioxidant activity (% inh.)		26.25 ± 2.05	19.24 ± 3.17	59.38 ± 1.05	88.70 ± 4.56
Total phenolic content (μg/g)		187.22 ± 8.64	106.67 ± 1.57	7511.11 ± 133.33	3023.89 ± 168.92
pH		5,6 ± 0,01	4,1 ± 0,02	7,4 ± 0,01	4.8 ± 0.02
Moisture (%)		17.8 ± 0.01	16.5 ± 0.01	82,5 ± 2,50	12,98 ± 1,02
Fat (%)		—	—	—	4,13 ± 0,09
Protein (%)		0.29 ± 0.003	0,18 ± 0,005	—	16,17 ± 0,02
Ash (%)		0,44 ± 0,11	0.13 ± 0.13	0.43 ± 0.17	1.60 ± 0.01
Color	L*	15,84 ± 7,49	31.66 ± 4.98	0.29 ± 0.10	63.58 ± 0.74
	a*	2.14 ± 1.89	1.47 ± 1.20	7.77 ± 1.69	16.22 ± 0.09
	b*	3.30 ± 1.58	4.14 ± 1.83	3.45 ± 0.61	67.69 ± 0.75
**(b)**					
			**BPM with chestnut honey**	**BPM with multifloral honey**	***p***
Antioxidant activity (% inh.)			86.30 ± 0.40	66.34 ± 3.03	0.013
Total phenolic content (μg/g)			1215.33 ± 49.08	780.33 ± 10.28	0,043
pH			5.20 ± 0.02	4.48 ± 0.01	0,530
Total titration acidity (meq/kg)			58,73 ± 2,49	60,99 ± 2,83	0,531
Moisture (%)			17.8 ± 0.01	15.8 ± 0.05	0,067
Fat (%)			0.19 ± 0.03	0.29 ± 0.04	0.186
Protein (%)			1.98 ± 0.03	1.77 ± 0.02	0.374
Ash (%)			0.870 ± 0.02	0.443 ± 0.05	0.030
Color		L*	49.61 ± 3.57	46.48 ± 1.96	0.542
		a*	12.91 ± 0.58	11.33 ± 1.67	0,224
		b*	41.69 ± 3.18	33.24 ± 3,07	0.229

For multifloral honey mixture;

$$\mathrm{AA}=+5.021+6.649^{*}A+4.619^{*}B$$

$$\mathrm{TPC}=+312.405+56.485^{*}A+34.006^{*}B$$

$$\mathrm{TMC}=+0.114+0.015^{*}A+0.022^{*}B$$

$$\mathrm{OAS}=+4.661-0.122^{*}A-0.059^{*}B$$

For chestnut honey mixture;

$$\mathrm{AA}=+22.22+6.504^{*}A+3.580^{*}B$$

$$\mathrm{TPC}=+418.392+96.795^{*}A+31.975^{*}B$$

$$\mathrm{TMC}=+0.466+0.006^{*}A+0.038^{*}B$$

$$\mathrm{OAS}=+4.030-0.108^{*}A-0.0586^{*}B+0.014^{*}\mathrm{AB}-0.027^{*}A^{2}+0.003^{*}B^{2}$$

**FIGURE 1:**
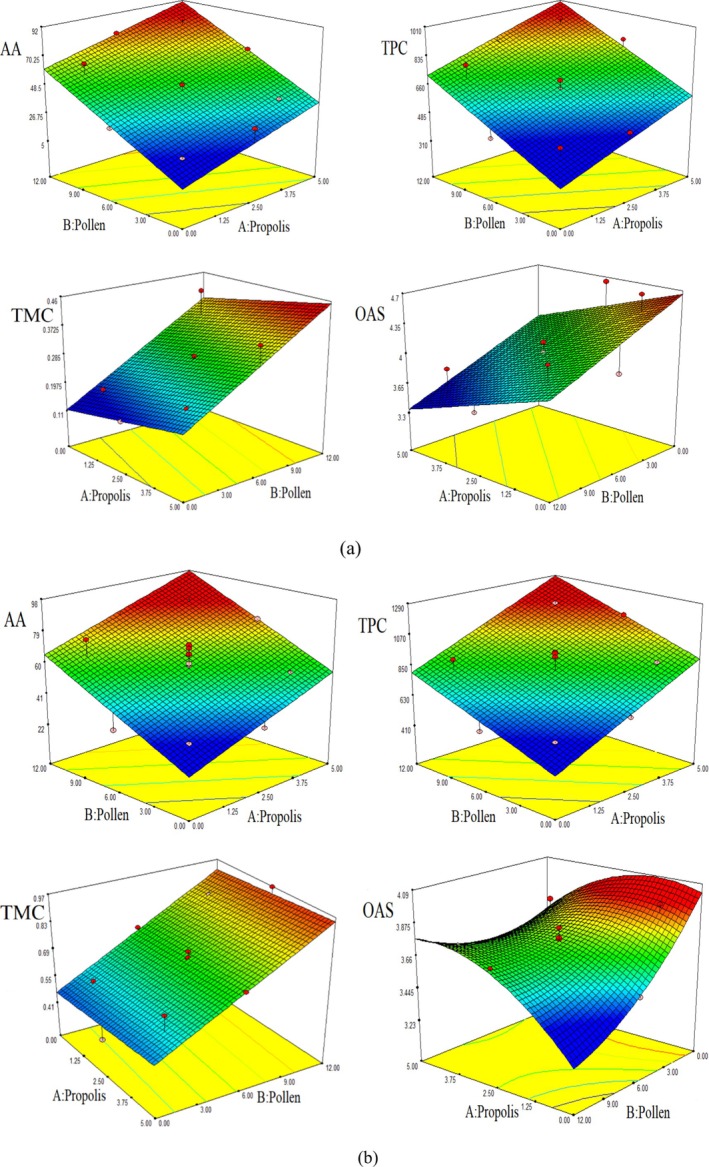
Response surface plots showing the mutual effects of the propolis (A) and pollen (B) usage amount on the AA, TPC, TMC, and OAS for multifloral honey (a), and chestnut honey–based mixture (b).

Figure 1a illustrates the interaction effect between the amounts of pollen and propolis on the responses (AA, TPC, TMC, and OAS) for the multifloral honey mixture. A linear function was deemed appropriate for AA, TPC, TMC, and OAS, as reflected in the graph (Figure 1a). The responses increased consistently with the rising amounts of pollen and propolis in the mixture.

Figure 1b shows the response of the interaction effect on the amount of propolis (A) and pollen (B) for the chestnut honey mixture. The TPC, AA, and TMC exhibited linear functions, and OAS exhibited quadratic functions. AA, TPC, and TMC increased with the amount of pollen and propolis. However, OAS tended to decrease after the central point with increasing independent factors.

The AA, TPC, TMC, and OAS were evaluated according to the desirability function, which was based on the idea that the “quality” of a product or process has multiple quality characteristics. The first solution had a desirability value (100%) from the suggested solutions by the software. This solution was selected as the optimum point and applied in the study (Table 1b).

According to the formulation optimization, a fixed 10.24% pollen content was used, along with a 1.73% propolis addition for the multifloral honey and 4.07% for the chestnut honey blend. In a study conducted by Habryka et al. (2020), propolis addition was tested in the range of 0.1%–1.0%, and while the bioactive properties were enhanced with propolis incorporation, a concentration of 0.3% was considered optimal due to undesirable taste and odor characteristics. Similarly, another study by Osés et al. (2016) found that enriching honey with propolis extract, obtained using 90% ethanol, improved bioactive properties; however, a 0.5% propolis addition was deemed suitable due to sensory limitations. In the present study, a glycerin‐based propolis extract was used, allowing for a higher proportion of propolis incorporation compared to previous studies. The findings suggest that the choice of extraction solvent significantly influences the amount of propolis that can be effectively incorporated into honey‐based mixtures.

### 3.2 Experimental Validation of the Optimization Results

The optimized levels in Table 1b were used to prepare the two mixture types in triplicate. The responses (AA, TPC, TMC, and OAS) of the mixture samples were analyzed for both honey types, and the average values of the results were calculated. Whether there was a statistically significant (*p* < 0.05) difference between the average and estimated values from the model by applying the one‐sample *t*‐test was evaluated. One‐sample *t*‐test results for each response are given in Table 1b. No statistical significance (*p* > 0.05) was detected between the results obtained from the validation. This result indicates that the model obtained via optimization was experimentally successful.

### 3.3 Characterization of Optimized Bee Product Mixture Samples

The mixture samples prepared by the optimized formulations were compared in terms of physicochemical and bioactive properties, amino acid, and mineral compositions.

#### 3.3.1 Physicochemical Properties

Some physicochemical properties of the bee products used in the mixtures and optimized mixture samples obtained from the formula optimization were determined and are presented in Table 2.

The physicochemical properties of the raw materials used in the bee product mixtures are presented in Table 2a. Pollen exhibited the highest values regarding antioxidant activity, fat, protein, and ash content, suggesting its significant potential to influence the characteristics of the food products it adds. Similarly, propolis showed high levels of total phenolic content and antioxidant activity. The elevated total phenolic content of propolis highlights its potential impact on the phenolic content and composition of the honey it combines with. When comparing the two types of honey, chestnut honey demonstrated higher antioxidant activity, phenolic content, protein, and ash content than multifloral honey. Honey contains a diverse range of natural antioxidant compounds, including phenolic acids, flavonoids, ascorbic acid, organic acids, proteins, carotenoids, and Maillard reaction products, as well as antioxidant enzymes such as catalase and glucose oxidase. These enzymes, inherently present in honey, contribute to its total antioxidant capacity (McKibben and Engeseth 2002). The phenolic composition of honey types depends on their botanical origin, leading to differences in their antioxidant activities. Furthermore, processing and storage conditions can influence honey's composition and antioxidant properties (Turkmen et al. 2005; Kamel et al. 2023).

The total phenolic content of the chestnut honey mixture was higher than the multifloral honey mixture, statistically significant (*p* < 0.05) (Table 2b). Similarly, the chestnut honey mixture showed significantly higher antioxidant activity and ash content (*p* < 0.05). The study conducted by Habryka et al. (2020) examined the effects of enriching honey with propolis on antioxidant activity and quality parameters. It was determined that propolis enhanced the honey's antioxidant activity and total phenolic content. This phenomenon may be attributed to the complex composition of bee products, as they are multifaceted natural substances with varying constituent profiles across different types. Consequently, these variations result from the presence of additional compounds that contribute to their overall antioxidant activity (Kamel et al. 2023). On the other hand, differences in moisture, total titratable acidity, pH, fat, protein, and color characteristics were also not statistically significant (*p* > 0.05).

#### 3.3.2 Phenolic Compounds

In this study, the phenolic compounds of the bee products and the optimized bee product mixtures (BPM) were characterized via LC–MS/MS, and the results are presented in Table 3.

**TABLE 3:** Phenolic compounds of raw materials and optimized bee product mixtures (BPM).

Phenolic compounds (ppb)	Pollen	Propolis	Multifloral honey	BPM with multifloral honey	Chestnut honey	BPM with chestnut honey
**Phenolic acids**						
Gallic acid	9527.88	1340.41	8557.37	8510.16	13512.54	9011.07
Cinnamic acid	—	70190.13	13368.07	10,425.23	3348.82	16501.68
Tannic acid	69.852,34	—	—	9.806,35	9.955,50	51282.53
Caffeic acid	24673.51	217751.56	25049.80	29615.15	23491.83	44121.57
2–5 dihydroxy benzoic acid	—	149.48	—	—	—	—
Trans ferulic acid	34439.37	709577.30	—	34402.08	31986.27	64134.30
CAPE	2046.74	369020.35	—	237.38	—	9278.81
Ellagic acid	208,137.20	3415.42	—	—	—	22795.68
**Flavonoids**						
*Flavonol*						
Quercetin	81591.86	1275.73	5815.16	11829.37	6336,82	18131.56
Rutin trihydrate	173,437.42	2271.69	—	19.755,32	—	35624.47
Myricetin	17802.47	1121.19	—	10662.78	10663.50	11649.41
*Flavan–3–ols*						
Catechin	7659.68	1257.17	1076.87	235.13	7880.12	5222.93
*Flavones*						
Chrysin	30093.16	98431.84	3760.51	15,816.40	4713.67	50719.15
Apigenin	254.16	52285.92	—	—	—	—
Luteolin	1905.29	—	—	—	—	—
*Flavanones*						
Naringenin	773710.71	32771.08	—	79192.56	—	122574.59
Total (ppm)	1435.13	1560,86	57.63	230.49	111.89	461.05

It was found that the propolis had the highest content of phenolic compounds (1560.86 ppm) among the bee products, followed by pollen (1435.13 ppm). Propolis has had the highest content of phenolic compounds (1560.86 ppm) among the bee products, followed by pollen (1435.13 ppm). Phenolic compounds, secondary metabolites of plant origin and found in many natural products, are agents responsible for antioxidant activity. At least 300 different compounds have been identified in propolis; their biological activities are attributed mainly to phenolic components, such as flavonoids (flavonols, flavones, flavanones, dihydro flavonols, and chalcones); aromatic aldehydes; terpenes; alcohols; and beta‐steroids. Phenolic compounds have biological activities, such as antioxidant potential, because they have hydroxyl groups and aromatic compounds in their chemical structures (Calegari et al. 2020).

Among the 16 phenolic compounds analyzed in this study, multifloral honey contained only six compounds, while chestnut honey contained nine. However, in terms of total content, chestnut honey had nearly double the phenolic compound content compared to multifloral honey. Multifloral honey had a total phenolic content of 57.63 ppm, whereas the BPM prepared with multifloral honey had 230.49 ppm. Similarly, chestnut honey had a total phenolic content of 111.89 ppm, while the optimized BPM reached 461.05 ppm. Enriching multifloral honey with pollen and propolis transformed it into a functional product regarding phenolic composition and content.

On the other hand, chestnut honey, already high in phenolic content, achieved even higher levels. The total amounts and the diversity of phenolic compounds in the mixtures were influenced, as shown in Table 3. For example, although multifloral honey did not naturally contain tannic acid, trans‐ferulic acid, CAPE, rutin trihydrate, myricetin, and Naringenin, these compounds were detected in the BPM with multifloral honey due to contributions from pollen and propolis. A similar situation was observed in the BPM with chestnut honey. Naringenin, a flavanone, was determined to be the most abundant compound in pollen (773.71 ppm). Although absent in both multifloral and chestnut honey, Naringenin was identified as the most abundant flavonoid in the BPM samples with multifloral honey (79.19 ppm) and chestnut honey (122.58 ppm). The study conducted by Habryka et al. (2020) examined the effects of enriching honey with propolis on antioxidant activity and quality parameters. The results revealed that the addition of propolis to honey increased the content of polyphenolic compounds, including flavonoids such as chrysin and pinocembrin, as well as phenolic acids like p‐coumaric acid and ferulic acid. This means that the difference in antioxidant activity is attributed to the different phenolic compounds in bee products. The high content of phenolic compounds and their diversity in pollen in propolis, which was reported by many researchers' investigations, reflected the high antioxidant activity (Kamel et al. 2023).

It was reported in several studies that the beneficial biological activities of propolis, such as antimicrobial, anti‐inflammatory, anti‐ulcer, and anticancer properties, are closely related to its bioactive compounds. While flavonoids are effective against various bacteria and protect against ulcers (Ruiz‐Hurtado et al. 2021), chrysin is known to have anti‐inflammatory and antineoplastic effects and functions as an important antioxidant and hepatoprotective agent (liver protector) (Shahbaz et al. 2023). Therefore, while the total phenolic content in the final product is significant, the diversity of phenolic compounds and their bioactive properties is equally important. These factors are the key determinants of the functional and nutritional value of the product.

#### 3.3.3 Amino Acid Composition

The amino acid composition in the optimized bee product mixtures identified via the LC–MS/MS method is listed in Table 4.

**TABLE 4:** Amino acid composition of raw materials and optimized bee product mixtures (BPM).

Amino acid (μmolar)	Pollen	Propolis	Multifloral honey	BPM with multifloral honey	Chestnut honey	BPM with chestnut honey
Leu*	131440.58	—	—	—	—	—
Ala	466595.81	608.56	3004.05	5878.58	2019,48	5270.09
Arg**	49374.15	1298.84	2611,54	2695.63	2610.75	2724.48
Asp	787878.66	—	881.84	4177,74	105,52	2.739,39
Cys**	12253.94	—	—	—	—	—
Gln	64224.23	—	—	—	—	—
Glu	742248.11	573.39	6793.76	15705.63	6480.02	10212.75
His*	51980.97	—	1308.04	1.768,20	1298.77	1669.55
İle*	105574.36	—	—	—	—	—
Lys*	42289.07	—	—	—	—	—
Met*	21825.73	—	—	—	—	—
Phe*	42569.20	—	599.39	633,57	—	—
Pro**	510355.92	354,93	30757.59	80140.47	22685,86	75104.56
Ser	325148.59	1614.83	4101.42	5395,55	3489.94	4686.53
Thr*	271763.83	258.10	1604.43	2686,21	1155,24	1493,69
Tyr**	27356,95	—	1900.30	1906,35	1836,08	1847,56
Val*	85833.26	—	—	—	—	—

*Note:* *Essential amino acids, **Semi‐essential amino acids.

All 17 amino acids analyzed in the study were found to be present in pollen. The presence of all essential amino acids, which cannot be synthesized by the human body, highlights the importance of pollen for human health. Notably, amino acids such as leucine (Leu), cysteine (Cys), glutamine (Gln), isoleucine (Ile), lysine (Lys), methionine (Met), and valine (Val), which are absent in other bee products, were exclusively found in pollen. Among the bee products, the 20% propolis extract used in the study contained the fewest types of amino acids (Ala, Arg, Glu, Pro, Ser, Thr), and their levels were also lower compared to other bee products.

While the amino acid compositions of multifloral honey and chestnut honey were similar, the levels in multifloral honey were generally higher. Additionally, phenylalanine (Phe) was exclusively detected in multifloral honey. The mixture samples also slightly reflected the amino acid profiles of the multifloral and chestnut honey used as raw materials. Phenylalanine was detected in the multifloral honey mixture, and the amino acid levels in the multifloral honey mixture were higher than those in the chestnut honey mixture. Since the amino acid levels in pollen are significantly higher than those in honey, it can be concluded that pollen is the primary determinant of the amino acid profiles in the mixture samples, whereas propolis has no significant effect.

#### 3.3.4 Mineral Composition

The chestnut honey used in the study was found to have a higher mineral content than the multifloral honey. The difference between Mn, K, and Ca elements was determined to be significant (*p* < 0.05). Manganese (Mn) was detected in chestnut honey but was absent in multifloral honey, which also influenced the composition of the mixtures. Mn was identified in the chestnut honey mixture but not in the multifloral honey mixture. Additionally, the K level in the chestnut honey mixture (3594.4 ppm) was higher than that in the multifloral honey mixture (983.00 ppm), and the difference was determined to be statistically significant (Table 5). The mineral content of honey varies depending on the nectar and pollen composition derived from plants. The primary mineral in honey is potassium (K), followed by calcium (Ca) and sodium (Na). The main trace elements include iron (Fe), copper (Cu), zinc (Zn), and manganese (Mn) (Kolaylı et al. 2008; Kaygusuz et al. 2016). Some studies have reported a correlation between the color of honey and its mineral content, with darker‐colored honey, like chestnut honey, typically having higher mineral levels (González‐Miret et al. 2005).

**TABLE 5:** Mineral contents of raw materials and optimized bee product mixtures (BPM).

Minerals (ppm)	Propolis	Pollen	Multifloral honey	BPM with multifloral honey	Chestnut honey	BPM with chestnut honey
Na	2338.33 ± 2.04^a^	23.60 ± 0.31^d^	11.21 ± 0.22^e^	54.49 ± 0.51^c^	16.02 ± 0.11^e^	102.52 ± 1.16^b^
Mg	2.77 ± 0.07^c^	59.13 ± 2.10^a^	2.62 ± 0.01^c^	11.14 ± 0.04^b^	5.24 ± 0.01^c^	12.61 ± 0.06^b^
Mn	0.01 ± 0.00^d^	1.42 ± 0.07^a^	—	0.21 ± 0.00^c^	0.92 ± 0.01^b^	1.36 ± 0.01^a^
Fe	—	4.06 ± 0.21^a^	—	—	—	0.14 ± 0.01^b^
Zn	—	0.45 ± 0.04^a^	—	0.13 ± 0.01^b^	—	0.12 ± 0.01^b^
P	—	116.09 ± 6.07^a^	5,13 ± 0,07^c^	18.61 ± 0.26^b^	5.65 ± 0.24^c^	21.28 ± 0.17^b^
K	55,9 ± 5,00^f^	3172.7 ± 32.4^b^	398.00 ± 2.8^e^	983.00 ± 6.1^d^	2166,20 ± 7,70^c^	3594.4 ± 0.03^a^
Ca	55.1 ± 0.50^f^	1182.3 ± 1.9^a^	103 ± 0,7^e^	229.00 ± 0.7^c^	198.10 ± 0.70^d^	314.7 ± 0.00^b^

*Note:* — Nondetermined. *Different letters (A–C) in the same line indicate the statistical significance (*p* < 0,05) of the difference between values.

The raw material containing all eight elements analyzed in the study was pollen. It was also determined to be the only raw material with the highest Ca, K, P, Mn, and Mg content. Additionally, pollen was the only bee product in the study that contained Fe and Zn. Sodium (Na), on the other hand, was found to be highest in propolis (2338.33 ppm), and its addition to the optimized mixtures increased the Na content of those mixtures. When comparing the mineral compositions of honey and the bee product mixtures (BPM) produced, a significant enrichment in elemental content in the mixtures was observed. Between the two mixtures, the chestnut honey BPM had a richer composition than the multifloral honey BPM. While the differences in Na, Mn, K, and Ca elements were statistically significant (*p* < 0.05), those in Mg, Fe, Zn, and P were insignificant (*p* > 0.05).

Honey is rich in minerals such as iron, phosphorus, magnesium, manganese, sodium, and iodine, which are essential for the human body (Mutlu et al. 2017). Its mineral content varies depending on the plant's nectar yield and pollen composition. Trace elements function as cofactors for numerous antioxidant and non‐antioxidant enzymes. Therefore, minerals are vital components of metabolism and the overall integrity of the human body (Bakour et al. 2022). The enrichment of mineral content in the optimized BPM samples obtained in this study compared to the honey samples indicates that the products have been elementally fortified and have gained enhanced functionality.

## 4 Conclusion

Recent studies and consumer preferences have focused on uncovering the functional properties of bee products, emphasizing their use as dietary supplements and for apitherapeutic purposes, and they have shown that these products exhibit complementary nutritional and bioactive effects, including antioxidant, antimicrobial, anti‐inflammatory, and immunomodulatory activities individually. On the other hand, the combination of bee‐derived products, such as honey, pollen, and propolis, presents a promising approach to enhancing their functional and health‐promoting properties. This study aimed to transform chestnut honey, commonly used for apitherapeutic purposes and holding commercial significance, and multifloral honey, frequently consumed in daily diets, into functional final products by combining them with other bee products and characterizing their properties. For this aim, two types of honey‐based bee‐product mixture formulations were optimized, and they were compared in terms of physicochemical and bioactive properties, amino acid, and mineral compositions. As confirmed by the results of this study, especially chestnut honey provides a rich source of phenolic compounds and flavonoids, pollen contributes essential amino acids, minerals, and bioactive properties, while propolis is characterized by its high polyphenol content. In this study, the synergistic interaction of these components was presented in terms of some bioactive, nutritional, physicochemical, and sensory properties. The optimized bee product mixtures demonstrated significantly higher phenolic content, amino acid composition, and mineral profiles than the two types of honey used as raw materials (chestnut and multifloral honey). Furthermore, it can be stated that pollen is the primary bee product that determines the functional properties of the final product mixtures. Therefore, formulating a single functional mixture incorporating these three bee products offers a novel and effective strategy for maximizing their beneficial properties while improving consumer accessibility to high‐quality, safe bee‐derived functional foods. While these results provide general insights into their functionality, further advanced clinical studies are necessary to draw definitive conclusions regarding their apitherapeutic properties. It should be studied more deeply such as their bioavailability, overall efficacy in promoting health, and preventing oxidative stress‐related diseases.

## Author Contributions

**Melike Ağirsaygin:** formal analysis (equal), resources (equal). **Müge Hendek Ertop:** conceptualization (equal), data curation (equal), funding acquisition (equal), project administration (equal), supervision (equal), writing – original draft (equal), writing – review and editing (equal).

## Ethics Statement

The authors have nothing to report.

## Consent

The authors have nothing to report.

## Conflicts of Interest

The authors declare no conflicts of interest.

## Data Availability

The Data that support the findings of this study are available on request from the corresponding authors.
